# Quality of mental health questionnaires in conflict-affected adult populations in low and middle income countries: A systematic review

**DOI:** 10.1016/j.jmh.2021.100068

**Published:** 2021-10-08

**Authors:** Sharon Christy, Chesmal Siriwardhana, Julia Lohmann, Bayard Roberts, Sarah Smith

**Affiliations:** a London School of Hygiene and Tropical Medicine, Keppel St, London WC1E 7HT, UK; b Institute of Global Health, Heidelberg University Hospital, Im Neuenheimer Feld 130.3, Heidelberg 69120, Germany

**Keywords:** Global mental health, Psychometrics, Validation study, Diagnosis, Mental health screening, War

## Abstract

**Background:**

Accurate measurement of mental health disorders in conflict-affected populations is crucial for improving mental health care for these populations. Most studies to develop mental health questionnaires for conflict-affected populations are conducted in high income countries despite the vast majority of conflict-affected populations residing in Low and Middle Income Countries (LAMICs). The aim of this systematic review is to assess the quality of questionnaires for mental disorders that have been either developed or validated in conflict- affected settings in LAMICs.

**Methods:**

A systematic review of 5 databases (CINAHL Plus, EMBASE, Global Health, MEDLINE and PsycINFO) was conducted to identify validation studies for questionnaires measuring mental health disorders in adult conflict-affected population in LAMICs. Well-established psychometric criteria evaluating reliability, validity and responsiveness of questionnaires were applied for quality appraisal.

**Results:**

Thirty validation studies were included in this review, which reported on data for 33 questionnaires. Twenty-four were questionnaires that had been originally developed in different settings and adapted for use with a new conflict-affected population and 9 had been newly developed for the conflict-affected population being studied. Overall, there was high variability in the quality of evidence for the questionnaires with moderate evidence for the validity and reliability of included questionnaires but no responsiveness data reported.

**Conclusion:**

There has been increasing recognition of the particular importance of psychometrics in this field to facilitate the development of good quality mental health questionnaires suitable for use in LAMICs. However, this review highlighted the current limited quantity and quality of such questionnaires.

## Highlights

• Quality is variable for conflict-affected populations’ mental health questionnaires
• We found moderate evidence for reliability and validity but none for responsiveness
• Equity in authorship and populations covered must be improved
• Research capacity in conflict-affected settings needs strengthening
• We recommend stronger use of conceptual frameworks and reporting standards

## 1 Introduction

An estimated 172 million people are affected by armed conflict worldwide, including over 59 million people forcefully displaced from their homes either within their countries as internally displaced persons (IDPs) or into new countries as refugees. (Centre for Research on the Epidemiology of Disasters, 2013) Conflict is associated with increases in both physical and mental health needs coupled with the breakdown of health systems. (Silove et al., 2017; Spiegel et al., 2010; Roberts and Browne, 2011) Mental health disorders are more prevalent among populations exposed to conflict; a systematic review and meta-analysis on prevalence estimates of mental disorders in conflict-affected settings found that the estimated total prevalence of depression, anxiety, post-traumatic stress disorder, bipolar disorder, and schizophrenia was 22·1% (95% UI 18·8–25·7). (Charlson et al., 2019) Poor mental health among conflict-affected populations is related to exposure to violent and traumatic events, forced migration, increased daily stressors related to poverty, unemployment, and social isolation. (Porter and Haslam; 2015; Steel et al., 2009; Miller and Rasmussen, 2010) However, it is also important to recognise that the majority of conflict-affected people do not have mental health disorders and their resilience may be supported by protective factors such as high quality social support, family support and appropriate coping strategies. (Siriwardhana et al., 2014; Seguin and Roberts, 2017).

A pre-requisite for generating good quality evidence for addressing the mental health needs of conflict-affected populations is having good quality questionnaires to measure the mental health status of people in these situations. Some questionnaires have been developed for general use and are widely used in many different settings globally (e.g. Hopkin's Symptom Checklist) whereas others have been designed specifically for conflict-affected populations (e.g. Harvard Trauma Questionnaire). The latter are arguably likely to be more sensitive and relevant for use with conflict-affected populations. However, general mental health measures can also be used with conflict-affected populations if they have been validated appropriately. Expert consensus has prioritised the need to strengthen the evidence base for appropriate methods to assess the mental health and psychosocial needs of populations in humanitarian settings to improve mental health and psychosocial support in humanitarian settings. (Tol et al., 2011) Collecting health data on conflict-affected populations is challenging for reasons such as security risk posed to researchers and participants in collecting data, highly mobile populations necessitating rapid data collection methods and impeding follow-up, limited resources and capacity, and ethical concerns. (Siriwardhana et al., 2013; Blanchet et al., 2017; Checchi et al., 2017) These factors can make it difficult to collect data on mental health and hinder the development of mental health questionnaires specific to these contexts. Consequently, although the vast majority of conflict-affected populations reside in low and middle income countries (LAMICs), (Internal Displacement Monitoring Centre, 2015; United Nations High Commissioner for Refugees, 2014) questionnaires to measure mental health are mostly developed in English-speaking high-income countries and based on the understanding of mental health that is prevalent in these countries.

Meta-analyzes of the prevalence of PTSD and depression in conflict-affected populations have found that a large proportion of the variation in results between studies arose due to methodological factors such as the choice of questionnaires. (Charlson et al., 2019; Steel et al., 2009; Fazel et al., 2005) Evidence in LAMICs (albeit not with conflict-affected populations) suggests that questionnaires are often not appropriately validated before their use. (Tsai et al., 2013; Tsai, 2014) A systematic review from 2002 on health status questionnaires used with refugees identified 183 papers and found that measurements were mainly derived from, “instruments that have limited or untested validity and reliability in refugees.” (Hollifield et al., 2002) However, this review was for refugees only and dominated by studies in high-income countries. There has also been a very large increase in the number of mental health papers published with conflict-affected populations since 2002. (Blanchet et al., 2017)

To date, there have not been any systematic reviews published on the suitability and appropriateness of mental health questionnaires that are developed or evaluated for conflict-affected populations in LAMICs. The aim of this systematic review is to assess the quality of questionnaires for mental disorders that have either been developed or validated in conflict- affected settings in LAMICS.

## 2 Methods

### 2.1 Search strategy and selection criteria

The systematic review method followed PRISMA guidelines (Moher et al., 2009).

The databases searched were CINAHL Plus, EMBASE, Global Health, MEDLINE and PsycINFO. The initial search was carried out on 12^th^ August 2016 and then updated on 16^th^ October 2019. The search included all the articles published from the inception of each database to the last search date.

Search terms were developed for three concepts: measurement properties, mental health and armed conflict. The search was conducted using search filters coupled with a comprehensive set of free search terms and index terms from the Consensus-based Standards for the Selection of Health Measurement Instruments (COSMIN) guidelines. (Terwee et al., 2009) The full search terms are given in the online supplementary materials (Appendix A). The reference lists of the studies included in the review were also manually searched.

### 2.2 Inclusion criteria

The population of interest was civilian adults (aged 18+ years) in LAMICs either forcibly displaced by conflict within their own country (IDPs) or outside of their own country (refugees) following standard definitions (Roberts and Browne, 2011; Deng, 1998; United Nations, 1951) and people currently living in a conflict-affected area or one affected by conflict within the last 5 years (including returned IDPs and refugees). Armed conflict was defined as “a contested incompatibility which concerns government and/or territory where the use of armed force between two parties, of which at least one is the government of a state, results in at least 25 combatant battle-related deaths per year.” (Uppsala University, 2015)

The primary aim of included studies had to be to develop a mental health questionnaire or evaluate the measurement properties of a pre-existing questionnaire in a conflict setting. A questionnaire was considered a unique questionnaire if it had been newly developed for a conflict-affected population or if it had been adapted for a new conflict-affected population.

Articles were included if they reported at least one measurement property of a self-reported questionnaire measuring a specific mental health disorder as defined in an edition of the International Classification of Disease (ICD) or the Diagnostic and Statistical Manual (DSM) or a generic questionnaire with a specifically-identified cut-off point for a diagnosable disorder.

Only studies published in a peer-reviewed journal in English or French were included.

### 2.3 Exclusion criteria

Studies including study participants primarily displaced due to reasons other than conflict (e.g. natural disasters) and war combatants and military veterans were excluded.

Studies that included results from validating a questionnaire but did not have validation as a primary aim were excluded as many of these studies did not present adequate information about the validation methods for quality appraisal.

Studies on questionnaires measuring general psychological health and mental distress were excluded to focus on how suitable existing questionnaires are for detecting mental health disorders recognised in international classifications. Results from studies describing assessments that were based only on clinical-rating scales, interviews, group discussions, performance-based tests, diaries, videos, telephone calls, laboratory tests, or imaging were also excluded.

### 2.4 Data extraction

Retrieved articles were transferred to Mendeley Version 1.19.4. Duplicates were removed and titles and abstracts were screened. For those studies appearing to meet the inclusion criteria, the full text was retrieved for confirmation. For queries about whether papers met the inclusion criteria that could not be resolved on review of the full text, the authors were contacted for clarification.

For included articles, data about the measurement properties of each questionnaire were extracted using a standard data extraction form and compiled into tables. For the questionnaires that had originally been developed in different settings, the *adapted* questionnaires, the original development papers were then searched for. The data from these original development papers were compiled into a separate table for comparison with the results from the new conflict-affected settings. The search strategy, study selection and data extraction were carried out by one of the authors (SC) with any queries discussed with two of the other authors (BR and SS).

### 2.5 Critical appraisal

Psychometric properties and criteria for quality appraisal within the Classical Test Theory paradigm are based on well-established psychometric guidelines to evaluate reliability, validity and responsiveness (Scientific Advisory Committee of the Medical Outcomes Trust, 2002; Guidance for Industry, 2006; Reeve et al., 2013) as used by Protopapa et al. (2017) (Table 1). These quality appraisal criteria were applied to all the questionnaires identified through the search. Quality appraisal criteria were applied to the data collected from the study population under investigation for each unique questionnaire. For the adapted questionnaires, the quality appraisal criteria were also applied to their parent questionnaires using the data from their original development paper(s). The available evidence for each psychometric property for each questionnaire was rated on a 4-point ratings scale (no evidence; limited evidence; moderate evidence; strong evidence).

**Table 1:** Quality appraisal criteria for questionnaires.

Psychometric property	Definition/test	Criteria for acceptability
1. Reliability		
1.1 Internal consistency	The extent to which items comprising a scale measure the same construct (e.g. homogeneity of the scale); assessed by Cronbach's a	Cronbach's αs for summary scores ≥0.70
1.2 Test-retest	The stability of a measuring instrument; assessed by administering the instrument to respondents on two different occasions and examining the correlation between test and retest scores	Test–retest reliability correlations for summary scores ≥0.70
1.3 Inter-rater	The extent to which scores for patients who have not changed are the same for repeated measurement by different persons	Inter-rater reliability correlations ≥0.70
2. Validity		
2.1. Content validity	The extent to which the content of a scale is representative of the conceptual domain it is intended to cover; assessed qualitatively during the questionnaire development stage through pre-testing with patients, expert opinion and literature review	Qualitative evidence from pre-testing with patients, expert opinion and literature review that items in the scale are representative of the construct being measured
2.2. Criterion-related validity		
2.2.1 Concurrent validity	Evidence that the scale predicts a ‘gold standard’ criterion that is measured at the same time; assessed on the basis of correlations between the scale and the criterion measure	High correlation between the scale and the criterion measure
2.2.2 Predictive validity	Evidence that the scale predicts a ‘gold standard’ criterion that is measured in the future; assessed on the basis of correlations between the scale and the criterion measure.	High correlation between the scale and the criterion measure
2.3 Construct validity		
2.3.1 Within-scale analyzes	Evidence that a single entity (construct) is being measured and that items can be combined to form a summary score; assessed on the basis of evidence of good internal consistency and correlations between scale scores (which purport to measure related aspects of the construct)	Internal consistency (Cronbach's a) ≥0.70. Moderate to high correlations between scale scores Adequate factor analysis
2.3.2 Analyzes against external criteria		
2.3.2.1 Convergent validity	Evidence that the scale is correlated with other instruments measuring the same or similar constructs; assessed on the basis of correlations between the instrument and other similar instruments	Correlations are expected to vary according to the degree of similarity between the constructs that are being measured by each instrument Specific hypotheses are formulated and predictions tested on the basis of correlations.
2.3.2.2 Discriminant validity	Evidence that the scale is not correlated with instruments measuring different constructs; assessed on the basis of correlations with instruments measuring different constructs	Low correlations between the instrument and instruments measuring different constructs
2.3.2.3 Known groups differences	The ability of a scale to differentiate known groups; assessed by comparing scores for subgroups who are expected to differ on the construct being measured	Significant differences between known groups or difference of expected magnitude
2.3.2.4 Hypothesis testing	The extent to which the scale confirms pre-defined hypotheses regarding expected associations or lack of association with external factors, such as patient characteristics	Significant moderate to high correlations, or significant associations in the expected direction. Expected lack of association confirmed
3. Responsiveness	The ability of a scale to detect clinically important change over time; assessed by comparing scores before and after an intervention of known efficacy (on the basis of various methods including t-tests, effect sizes, standardised response means, or responsiveness statistics)	Significant differences between known groups or difference of expected magnitude.

Grading system for acceptability: 0 = no evidence in favour, + = limited evidence in favour, ++ = moderate evidence in favour, +++ = strong evidence in favour

Table adapted from Protopapa (2017) *Patient-reported outcome (PRO) questionnaires for men who have radical surgery for prostate cancer: a conceptual review of existing instruments* (Protopapa et al., 2017)

For the questionnaires identified through the search, the quality appraisal process was carried out independently by two of the authors (SC and JL) who then discussed any discrepancies with one of the other authors (SS) until reaching consensus. For the parent questionnaires of the adapted questionnaires, the quality appraisal process was carried out by one of the authors (SC) with any queries discussed with one of the other authors (SS).

## 3 Results

**Fig. 1:**
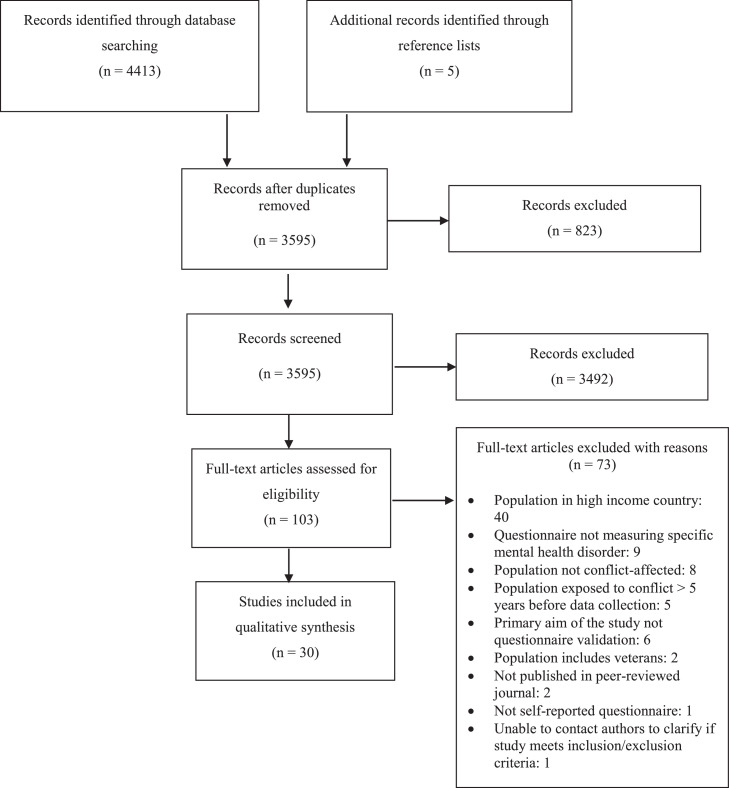
Study selection.

The study selection results are summarised in Fig. 1. The search returned 4413 results of which 823 were duplicates. Screening of titles and abstracts excluded a further 3492. Of the 103 full text articles assessed, the largest number were excluded for having a study population in a high-income country (*n* = 40) followed by the questionnaire not measuring a specific mental health disorder as defined in the ICD or DSM or being a generic questionnaire with no specifically-identified cut-off point for a diagnosable disorder (*n *= 9). Ultimately, 30 studies were included in the review. (Blair et al., 2017; Getnet and Alem, 2019; Ventevogel et al., 2007; Bolton, 2001; Michalopoulos et al., 2015; Tay et al., 2017; Tay et al., 2017; Dokkedah et al., 2015; Morina et al., 2013; Morina et al., 2010; Miller et al., 2009; Vallieres et al., 2018; Liddell et al., 2013; McDonald et al., 2019; Heeke et al., 2017; Ibrahim et al., 2018; Jayawickreme et al., 2012; Powell and Rosner, 2005; Vinson and Chang, 2012; Silove et al., 2017; Tay et al., 2018; Fellmeth et al., 2018; Tay et al., 2015; Tay et al., 2016; Tay et al., 2015; Veronese and Pepe, 2013; Ing et al., 2017; Farhood et al., 2015; Elsass et al., 2009; Tremblay et al., 2009) Of these studies, 18 had been published in the last 5 years (2015 onwards). (Blair et al., 2017; Getnet and Alem, 2019; Vallieres et al., 2018; McDonald et al., 2019; Ibrahim et al., 2018; Silove et al., 2017; Tay et al., 2018; Fellmeth et al., 2018; Tay et al., 2015; Tay et al., 2019; Tay et al., 2016; Tay et al., 2015; Ing et al., 2017; Farhood et al., 2015; Michalopoulos et al., 2015; Tay et al., 2017; Tay et al., 2017; Dokkedah et al., 2015)

Studies included study populations from a broad range of settings. These included: 7 African countries (Democratic Republic of Congo (Michalopoulos et al., 2015), Ethiopia (Getnet and Alem, 2019), Guinea (Vinson and Chang, 2012), Kenya (McDonald et al., 2019), Rwanda (Bolton, 2001), Sierra Leone (Vinson and Chang, 2012), and Uganda (2 studies) (Blair et al., 2017; Dokkedah et al., 2015)); 5 Asian countries (Afghanistan (2 studies) (Ventevogel et al., 2007; Miller et al., 2009), India (Elsass et al., 2009), Sri Lanka (2 studies) (Tay et al., 2017; Jayawickreme et al., 2012), the Thai-Myanmar border (3 studies) (Ing et al., 2017; Michalopoulos et al., 2015; Fellmeth et al., 2018) and Timor-Leste (2 studies) (Liddell et al., 2013; Tay et al., 2017)); 1 Oceanic country (Papua New Guinea (6 studies) (Tay et al., 2016; Tay et al., 2015; Tay et al., 2017; Tay et al., 2018; Tay et al., 2015; Tay et al., 2019)); 2 European countries (Bosnia-Herzegovina (Powell and Rosner, 2005) and Ex-Yugoslavia (2 studies) (Morina et al., 2013; Morina et al., 2010)); 3 Middle Eastern countries (Iraq (2 studies) (Michalopoulos et al., 2015; Ibrahim et al., 2018), Israeli-Palestinian conflict zone (Veronese and Pepe, 2013), and Lebanon (2 studies) (Farhood et al., 2015; Vallieres et al., 2018)); and 1 South American country (Peru (Tremblay et al., 2009)). Two studies included refugee participants in both high income countries (Germany, Italy and United Kingdom) and a LAMIC (Ex-Yugoslavia) (Morina et al., 2013; Morina et al., 2010) which provided disaggregated LAMIC data and so only the LAMIC-related data were included in the review.

The study populations were mainly refugees (16 populations) (Getnet and Alem, 2019; Tay et al., 2016; Vinson and Chang, 2012; Silove et al., 2017; Tay et al., 2018; Fellmeth et al., 2018; Tay et al., 2015; Tay et al., 2019; Tay et al., 2015; Ing et al., 2017; Elsass et al., 2009; Tremblay et al., 2009; Michalopoulos et al., 2015; Vallieres et al., 2018; McDonald et al., 2019; Ibrahim et al., 2018), followed by individuals living in post-conflict zones (10 populations) (Blair et al., 2017; Liddell et al., 2013; Tremblay et al., 2009; Bolton, 2001; Tay et al., 2017; Morina et al., 2013; Morina et al., 2010; Jayawickreme et al., 2012; Powell and Rosner, 2005; Silove et al., 2017), followed by those living in a conflict zone (6 populations) (Veronese and Pepe, 2013; Farhood et al., 2015; Ventevogel et al., 2007; Michalopoulos et al., 2015; Dokkedah et al., 2015; Miller et al., 2009), and the least frequently studied populations were IDPs (1 population) (Ibrahim et al., 2018).

Summary characteristics of the 33 questionnaires included in the review are presented in Table 2. Twenty four were questionnaires that had been originally developed in different settings and adapted for use with a new conflict-affected population (Blair et al., 2017; Getnet and Alem, 2019; Tay et al., 2017; Tay et al., 2017; Dokkedah et al., 2015; Morina et al., 2013; Morina et al., 2010; Miller et al., 2009; Vallieres et al., 2018; McDonald et al., 2019; Ibrahim et al., 2018; Powell and Rosner, 2005; Veronese and Pepe, 2013; Vinson and Chang, 2012; Fellmeth et al., 2018; Ing et al., 2017; Farhood et al., 2015; Elsass et al., 2009; Tremblay et al., 2009; Ventevogel et al., 2007; Bolton, 2001; Michalopoulos et al., 2015) and 9 had been newly developed for the conflict-affected population being studied (Liddell et al., 2013; Tay et al., 2016; Tay et al., 2015; Tremblay et al., 2009; Tay et al., 2017; Jayawickreme et al., 2012; Tay et al., 2018; Tay et al., 2015; Tay et al., 2019).

**Table 2:** Summary characteristics of the questionnaires included in the review.

Questionnaire name, reference papers/manuals	Mental health construct	Description of items and domains	Adaptations made from original questionnaire	Response options and scoring	Target population (language), recall period
AUDIT (Blair et al., 2017)	Alcohol use disorders	10 items 3 domains: (1) Hazardous consumption (items 1-3) (2) Alcohol dependency (items 4-6) (3) Alcohol-related physical, mental and social harms (items 7-10)	Items translated and back translated into Acholi Luo then piloted	Responded on a 5-point Likert scale apart from the last 2 items which were scored on a 3-point scale Potentially hazardous drinking defined as a score ≥1 on items addressing the number of drinks normally consumed Alcohol dependency defined as a score ≥ 1 on any of items 4 to 6 Alcohol-related harm defined as score >1 on any of the last 4 items	Post-conflict population in Northern Uganda (Acholi Luo), recall period not reported
CES-D (Getnet and Alem, 2019)	Depression	20 items 4 domains: (1) Positive affect (2) Negative affect (3) Somatic symptoms and retarded activity (4) Interpersonal difficulties	Already translated in previous studies	Responded on a 4-point Likert scale (0=none of the time, 3=most of the time) Scored by overall total (0-60)	Eritrean refugees living in the Mai-Aini refugee camp, Northern Ethiopia (Trigringa), 1 week
Community-based anger measure (Liddell et al., 2013)	Intermittent explosive disorder (IED)	10 items 7 domains: (1) Descriptors of anger attacks (2) Triggers and the contextual inappropriateness of anger attacks (3) Level of controllability of anger (4) Frequency of attacks (5) Manifestations of aggressive behavior (6) Physiological manifestations of anger (7) Associated psychosocial impairment	Not applicable as newly developed questionnaire	6 items: a visual analogue scale of 7 circles increasing in size and darkness to indicate increasing severity 3 items: dichotomous responses (present/absent) 1 item: numerical response to the question ‘How often do the attacks occur?’ An algorithm was developed to score the items to yield a provisional IED diagnosis according to DSM-IV criteria	Individuals living in Timor-Leste in a post-conflict setting (Tetum), recall period 1 month (for 1 item) but not reported for other items
Culturally adapted checklist for complicated grief (later developed into the complicated bereavement module of the R-MHAP) (Tay et al., 2016)	Complicated grief	18 items	Not applicable as newly developed questionnaire	Not reported	West Papuan refugees living in Papua New Guinea (Baha Indonesian), since the death or loss of a family members and/or close friend in the last 12 months
Complicated bereavement module of the R-MHAP (Tay et al., 2019)	Complicated bereavement	18 items	Identical to the above questionnaire apart from item 18 changed from “*Had difficulty or been reluctant to plan for the future or pursuing other interests since the person's death”* to “*Had difficulty or been reluctant to plan for the future”*	Responded on a 4-point Likert scale (1=not at all, 4=extremely) To make a provisional diagnosis of complicated bereavement, the ordinal scale was collapsed into a categorical response through a symptom being regarded as present if scored as either 3 or 4	West Papuan refugees living in Papua New Guinea (Baha Indonesian), since the death or loss of a family members and/or close friend in the last 12 months
Culturally adapted checklist for PTSD and CPTSD (Tay et al., 2015)	CPTSD PTSD	21 items	Not applicable as newly developed questionnaire	Responded on a dichotomous scale (present/absent) Diagnosis made based on algorithms derived from DSM-IV/5 and ICD 10/11 definitions of PTSD and CPTSD	West Papuan refugees living in Papua New Guinea (Baha Indonesian), recall period not reported
CRIES-13 (Veronese and Pepe, 2013)	PTSD	13 items 3 domains: (1) Intrusion (4 items) (2) Avoidance (4 items) (3) Arousal (5 items)	Already translated into Arabic in previous studies	Responded on a 4-point Likert scale (not at all, rarely, sometimes, often; scores 0, 1, 3, and 5 respectively) Scored by overall total (0-65)	Adult Arab NGO workers working in the Israeli-Palestinian conflict zone (Arabic), recall period not reported
EPDS (Ing et al., 2017)	Postnatal depression	10 items	Translation and back-translation	Responded on a 4-point Likert scale (0–3) Scored by overall total (0-30) with higher scores indicating more symptoms	Postpartum migrant and refugee women on the Thai–Myanmar border (Karen Burmese), 1 week
GHQ-28 (Farhood et al., 2015)	Common mental disorders (with a specific cut-off point for depression)	28 items 4 domains (7 items each): (1) Somatic symptoms (2) Anxiety and insomnia (3) Social dysfunction (4) Severe depression	Already translated into Arabic in a previous study Scoring for the severe depression domain adapted as described in the following column	Responded on a 4-point Likert scale of (0-3, indicating never, same as usual, more than usual, a lot more than usual respectively) Responses of 0/1 assigned a score of 0 Responses of 2/3 assigned a score of 1 Scored for each domain For the severe depression domain, the above scoring system did not yield meaningful cut-off points so the scores were recalculated based on the original 4-level ordinal scale responses	General population living in Southern Lebanon during conflict (Arabic), recall period not reported
HSCL-25 (Elsass et al., 2009)	Anxiety Depression	25 items 2 domains: (1) Anxiety (2) Depression	Translated and back-translated with focus group discussion then pilot-testing	Responded on a 4-point Likert scale according to symptom severity Score calculated by dividing the total score by number of items answered	Tibetan refugees enrolled in the Tibetan Torture Survivor Programme living in Dharamasala, India (Tibetan), 1 week
HSCL-25 (Tremblay et al., 2009)			Translated and back-translated	Response options and detailed scoring methods not reported Score of 1.75 defined as a cut-off point for both depression and anxiety, and for a combined total response	Individuals living in the Peruvian rural highlands and northern Ayacucho (urban Peruvian setting) who had been affected by the Peruvian civil conflict and were either returnees, refugees or living in post-conflict settings (Quechua and Spanish), recall period not reported
HSCL-25 (Ventevogel et al., 2007)			Translated and back-translated with focus group discussion Due to low levels of literacy, questionnaire administered by a trained lay interviewer	Responded on a 4-point Likert scale from 1 (not at all) to 4 (extremely) Score calculated by dividing the total score by number of items answered to generate an anxiety and a depression score ranging from 1 to 4	Pashtuns living in Eastern Afghanistan during the conflict attending for primary care services (Pashto), 1 month
HSCL-depression subscale (Bolton, 2001)	Depression	18 items	Translation, back-translated and edited by a local expert panel (1) Items added to cover locally relevant symptoms (loss of intelligence, mental instability, and loss of trust in others) (2) Item added on psychomotor agitation to improve consistency with DSM criteria and because this symptom was reported locally (3) Item on "feeling trapped" was removed as this did not conform with DSM criteria and was not mentioned locally	Responded on a 4-point Likert scale (1= no symptoms, 4= severe symptoms) Scored by overall total	Post-conflict population living in rural areas near Kigali, Rwanda (Kinyarwanda), recall period not reported
HTQ (adapted for the DSM-4) (Michalopoulos et al., 2015)	PTSD	16 items	Original 5 response categories reduced to 4 as described in the following column	In the DRC and Iraq, there were 4 response categories for each item of the HTQ because during the translation and validation it was clear that the language did not have distinctions between 5 response categories In Burma, there were originally five response categories (0=none of the time, 1=a little of the time, 2=some of the time, 3=most of the time, 4=almost all the time) but, for consistency across the samples, the Burma HTQ items were collapsed to 4 response categories by combining the two highest response options Scored by overall total	3 different populations: (1) Kurdish torture survivors living in a conflict zone in Northern Iraq (2) Female sexual violence survivors living in a conflict zone in Eastern Democratic Republic of Congo (DRC) (3) Burmese refugees in Thailand at the Thailand-Myanmar border (languages not reported), 1 week
HTQ (adapted for the DSM-5) (Michalopoulos et al., 2015)		20 items	Original 5 response categories reduced to 4 as described in the following column For the DSM-5 model, 4 additional items were used: (1) Blaming yourself for things (2) Feeling guilty (3) Feeling shame (4) Drinking too much alcohol* *In Burma, there was not a ‘drinking too much alcohol’ item or other proxy item that was felt representative of reckless or self-destructive behavior so this item was not included in the analysis for Burma.		
HTQ (Tay et al., 2017)		24 items: 16 items from the original HTQ 8 additional items as previously identified to be relevant to the local population	HTQ previously translated into Tamil HTQ translated and back-translated into Sinhalese Addition of 8 items identified to be relevant locally	Responded on a 4-point Likert scale (1=not at all, 2=a little, 3=quite a lot, 4=extremely) Due to the generally low endorsement of symptoms, the scored items were grouped according to a binary format (0 = not at all or; 1 = a little/quite a bit/extremely) for analysis	Post-conflict general population living in Sri Lanka (Tamil and Sinhalese), recall period not reported
HTQ (Tay et al., 2017)		17 items	‘Refined items to ensure their cultural, semantic and linguistic appropriateness when translated and applied in Timor-Leste’ Included an additional symptom of ‘physiological reactivity in response to reminders of the trauma’ to reflect the DSM-IV criteria	Responded on a 4-point Likert scale (1 =none, 4=most of the time)	Post-conflict general population in Dili (capital of Timor-Leste) and a rural site 1 h drive away (Tetum), recall period not reported
ICD11- Trauma Questionnaire for CPTSD (Dokkedah et al., 2015)	CPTSD	17 items 4 domains: (1) Emotional regulation of hyperactivation (2) Emotional regulation of deactivation (3) Negative self-concept (4) Disturbed relationships	Translated and back-translated	Responded on a 5-point Likert scale (0-4) Each domain has a different threshold, which needs to be fulfilled to receive the diagnosis of C-PTSD Can only meet criteria for CPTSD if criteria met for PTSD (as per questionnaire in row below)	General population living in Gulu (Northern Uganda) during the Ugandan Civil War (Luo), recall period not reported
ICD-11 Trauma Questionnaire for PTSD (Dokkedah et al., 2015)	PTSD	7 items 3 domains: (1) Re-experiencing the traumatic event (2) Avoidance (3) Hyper-vigilance	Translated and back-translated	Responded on a 5-point Likert scale (0-4) Each domain needs at least one items score > 2 to fulfil the PTSD diagnosis	
IES-R (Morina et al., 2013)	PTSD	22 items 3 domains: (1) Intrusion (2) Hyperarousal (3) Avoidance	Previously translated for research in Ex-Yugoslavia	Responded on a 5-point Likert scale (0=not at all, 4=extremely) Scored by overall total and for each domain	2 study populations: (1) General population living in post-conflict settings in Ex-Yugoslavia (Bosnia-Herzegovina, Croatia, Kosovo, Macedonia, Serbia) (2) Refugees having been displaced to high income countries (HIC) (Germany, Italy, UK) by the war in Ex-Yugoslavia (language not reported), recall period not reported Results from HIC not included in quality assessment
IES-R (Morina et al., 2010)			Previously translated for research in Ex-Yugoslavia		2 study populations: (1) General population living in post-conflict settings in Ex-Yugoslavia (Bosnia-Herzegovina, Croatia, Kosovo, Macedonia, Serbia) (2) Refugees having been displaced to HIC (Germany, Italy, UK) by the war in Ex-Yugoslavia (language not reported), 7 days Results from HIC not included in quality assessment
IES-R (Miller et al., 2009)		23 items 3 sub-scales: (1) Intrusion (2) Hyperarousal (3) Avoidance	Translated and back-translated with group review process. An additional (23^rd^) item was added assessing the extent to which participants avoided talking about their symptoms of trauma in order to avoid upsetting others who might also be experiencing trauma symptoms (this item was only used descriptively and not included when calculating total IES-R scores for data analysis) Due to the low literacy rates, the items were read aloud to participants with responses as per the following column	A Likert-like scale using images of different levels of fluid in glasses with item choices ranging from 0 (empty glass/not at all) or 4 (full glass/extremely) Total scores (excluding the 23^rd^ item response) used for data analysis	General population living in Kabul (Afghanistan) in conflict zone (Dari), 1month
International Trauma Questionnaires (Vallieres et al., 2018)	CPTSD PTSD	18 items 2 domains each with 6 items: (1) Re-experiencing, avoidance, threat (2) Disturbances in self-organisation 6 further items to measure functional impairment associated with PTSD and disturbances in self-organisation symptoms	Translated and back-translated	Responded on a five-point Likert scale (0=not at all, 4=extremely) PTSD defined as scoring ≥2 for at least one item in each domain plus scoring ≥1 for at least one functional impairment item CPTSD defined as meeting PTSD scoring criteria and the following scores in the disturbances in self-organisation domain: 1. affective dysregulation-hyperactivity ≥10 2. affective dysregulation-hypoactivity ≥8 3. negative self-concept ≥8 4. disturbances in relationships ≥6	Syrian refugees living in Lebanon seeking mental health and psychosocial support (Arabic), 1 month
PCL-17-C (McDonald et al., 2019)	PTSD	17 items 3 domains: (1) Re-experiencing (2) Avoidance (3) Hyperarousal	Translated and back-translated Response options were modified to reflect styles of responding (a 5-point Likert scale was presented with five images of glasses with varying levels of water) Soring adapted as described in the following column	Responded on a five-point Likert scale (0=not at all, 1=rarely, 2=sometimes, 3=often, 4=almost always) Scored by overall total and for each domain For analysis, the 0–4 scale was collapsed by combining categories 1 and 2, yielding a scale of 0–3	Somali refugees in Nairobi's Eastleigh Estate, Kenya (Somali and English), recall period not reported
PCL-5 (Ibrahim et al., 2018)	PTSD	20 items 3 domains: (1) Intrusion (2) Avoidance (3) Negative alterations in cognition and mood (4) Hyperarousal symptoms	Translated and back-translated with focus group discussions	Responded on a five-point Likert scale, (0=not at all, 4=extremely) Scored by sum of all items (0-80)	Iraqi IDP and Syrian refugees living in the Kurdistan region of Iraq (Arabic, 2 Kurdish dialects: (1) Sorani (2) Kurmanji) Recall period not reported
PRP-WPQ (Jayawickreme et al., 2012, Jayawickreme et al., 2009)	Anxiety Depression Other psychological problems	164 items 3 domains: (1) Trauma exposure (22 items) with 2 subsections: torture and other war trauma (2) War-related general problems (84 items) with 5 subsections: family problems, economic problems, social problems, lack of basic needs, and physical problems (3) War-related psychological and behavioral problems (58 items) with 3 subsections: anxiety, depression, and other psychological problems	Only used the trauma exposure and war-related psychological and behavioral problems sections of the original questionnaire	Trauma exposure domain: respondents indicated whether they have experienced the trauma in question +/- the number of times they had experienced that trauma War-related psychological and behavior problems section: responded on a 4-point Likert scale (1=not at all, 4=extremely) Scored by total for each domain	Individuals receiving psychosocial assistance at clinics living in post-conflict setting in North-eastern Sri Lanka (Tamil), recall period not reported
PTDS (Powell and Rosner, 2005)	PTSD	17 items 4 domains: (1) Traumatic events (2) The time of occurrence of the "most upsetting" event, together with the respondent's assessment of whether the event was life- threatening and whether it was accompanied by feelings of helplessness and intense fear (3) Re-experiencing, avoidance and arousal (4) The duration of the disturbance and the consequences for functioning	Translated and back-translated then pilot tested Replaced domain 1 items (traumatic events) with a checklist of traumatic events specific to the war in Bosnia and Herzegovina 1992–5 In some cases, interviewers had to read (+/- reformulate) some items due to low literacy levels	Responded on a five-point Likert scale, (0=not at all or once a month, 4=5 or more times a week/almost always) Scored by overall total and for each domain	General population living in a post-conflict setting after the Bosnian War in Bosnia-Herzegovina (Bosnian), recall period not reported
PTDS (Vinson and Chang, 2012)		17 items	Translated and back-translated	Responded on a 4-point Likert scale, (1=not at all,4=often) Scored by overall mean and mean for each of the items	Conflict-affected refuges living in refugee camps in Guinea or Sierra Leone from Sierra Leona, Liberia or Guinea attending mental health services within the camps (Kissi, Mende, Kono and Krio), recall period not reported
PTSD and CPTSD R-MHAP modules (Silove et al., 2017)	CPTSD PTSD	21 items	Not applicable as newly developed questionnaire	Not reported	West Papuans refugees in Port Moresby, Papua New Guinea (Bahasa Indonesian), recall period not reported
PTSD and CPTSD R-MHAP modules (Tay et al., 2018)				All items rated dichotomously (yes/no) Scoring not reported	West Papuans refugees in Kiunga, a town in the Western Province of Papua New Guinea (Bahasa Indonesian, English and Tok Pisin), recall period not reported
RHS-15 (Fellmeth et al., 2018)	Anxiety Depression PTSD	15 items	Burmese and Sgaw Karen translations by the RHS-15 authors	Items 1–14: responded on a 5-point Likert scale (0=not at all, 4=extremely) illustrated by a beaker filled to varying degrees. Item 15 is a distress thermometer which asks respondents to rate their level of distress (0=no distress, 10=extreme distress) Total score ≥12 on items 1–14 and/or score ≥5 on item 15 considered to be a positive score	Migrant women (labour migrants and refugees) living on the Thai-Myanmar border attending antenatal clinic (Burmese and Sgaw Karen), recall period not reported
R-MHAP (Tay et al., 2015)	Mental health module: Depression, generalized anxiety disorder, intermittent explosive disorder, panic disorder, persistent complex bereavement related disorder, psychosis, PTSD, separation anxiety disorder, somatic symptom disorder Alcohol and substance use module: alcohol and substance misuse	Mental health module: not reported Alcohol and substance use module: 5 items	Not applicable as newly developed questionnaire	Mental health module: not reported Alcohol and substance use module: items rated dichotomously (yes/no) Scoring: Mental health module: mean of all items for each specific disorder presented Alcohol and substance use module: not reported	West Papuan refugees living in Port Moresby, Papua New Guinea (Bahasa Indonesian and Pinyin) Recall period: Mental health module: current (last 12 months) and lifetime Alcohol and substance use module: not reported
Trauma Questionnaire (Tremblay et al., 2009)	PTSD	3 domains: (1) History of trauma (2) PTSD-related (3) Local idioms of distress	Not applicable as newly developed questionnaire	Response options not reported Scored by total for domains 2 and 3	Individuals living in the Peruvian rural highlands and northern Ayacucho (urban Peruvian setting) who had been affected by the Peruvian civil conflict and were either refugees or living in post-conflict settings (Quechua and Spanish), recall period not reported

AUDIT: Alcohol Use Disorders Identification Test, CES-D: Centre for Epidemiologic Studies Depression Scale; CPTSD: Complex posttraumatic stress disorder**;** CRIES-13: Children's Revised Impact of Events Scale-13; DSM: Diagnostic and Statistical Manual; EPDS: Edinburgh Postnatal Depression Scale**;** GHQ-28: General Health Questionnaire-28**;** HSCL-25: Hopkin's Symptom Checklist-25**;** HTQ: Harvard Trauma Questionnaire; ICD-11: International Classification of Disease-11; IES-R: Impact of Events Scale-Revised; PCL-17-C: Posttraumatic Stress Disorder Checklist – 17 – Civilian; PCL-5: Posttraumatic Stress Disorder Checklist for DSM-5; PRP-WPQ: The Penn/RESIST/Peradeniya War Problems Questionnaire; PTSD: Posttraumatic stress disorder; PTDS: Posttraumatic Stress Disorder Diagnostic Scale; R-MHAP: Refugee-Mental Health Assessment Package; RHS-15: Refugee Health Screener

The Hopkin's Symptom Checklist-25 (HSCL-5) was adapted in 4 studies (Elsass et al., 2009; Tremblay et al., 2009; Ventevogel et al., 2007; Bolton, 2001), the Harvard Trauma Questionnaire (HTQ) in 3 studies (Michalopoulos et al., 2015; Tay et al., 2017; Tay et al., 2017), the Impact of Events Scale – Revised (IES-R) in 3 studies (Morina et al., 2013; Morina et al., 2010; Miller et al., 2009), the PTSD Diagnostic Scale in 2 studies (Powell and Rosner, 2005; Vinson and Chang, 2012), and the complex post-traumatic stress disorder (CPTSD) and PTSD modules of the Refugee-Mental Health Assessment Package (R-MHAP) in 2 studies (Silove et al., 2017; Tay et al., 2018). Each of the other questionnaires was assessed in a single included study.

Most questionnaires (n=25) measured a single mental health disorder. Of the mental health disorders measured, PTSD was the disorder most frequently measured (20 questionnaires) (Tay et al., 2015; Veronese and Pepe, 2013; Vallieres et al., 2018; McDonald et al., 2019; Ibrahim et al., 2018; Powell and Rosner, 2005; Vinson and Chang, 2012; Silove et al., 2017; Tay et al., 2018; Fellmeth et al., 2018; Tay et al., 2015; Tremblay et al., 2009; Michalopoulos et al., 2015; Tay et al., 2017; Tay et al., 2017; Dokkedah et al., 2015; Morina et al., 2013; Morina et al., 2010; Miller et al., 2009), then depression (9 questionnaires) (Getnet and Alem, 2019; Farhood et al., 2015; Elsass et al., 2009; Tremblay et al., 2009; Ventevogel et al., 2007; Bolton, 2001; Jayawickreme et al., 2012; Fellmeth et al., 2018; Tay et al., 2015), then an anxiety or panic disorder (6 questionnaires) (Elsass et al., 2009; Tremblay et al., 2009; Ventevogel et al., 2007; Jayawickreme et al., 2012; Fellmeth et al., 2018; Tay et al., 2015), then CPSTD (5 questionnaires) (Tay et al., 2015; Dokkedah et al., 2015; Vallieres et al., 2018; Silove et al., 2017; Tay et al., 2018), then Complicated Grief/Prolonged Grief Disorder (3 questionnaires) (Tay et al., 2016; Tay et al., 2015; Tay et al., 2019), then Intermittent Explosive Disorder (Liddell et al., 2013; Tay et al., 2015) and alcohol or substance misuse (Blair et al., 2017; Tay et al., 2015) (2 questionnaires respectively). The remaining disorders (psychosis, postnatal depression and somatic symptom disorder) were measured by a single questionnaire (Ing et al., 2017; Tay et al., 2015). Of note, data for 8 of the 33 questionnaires included in this review were reported by the same set of collaborators with similar methods used for all of these studies. (Tay et al., 2016; Tay et al., 2015; Tay et al., 2017; Tay et al., 2017; Silove et al., 2017; Tay et al., 2018; Tay et al., 2015; Tay et al., 2019)

Results for the psychometric appraisal of the identified questionnaires are presented in Table 3. At least one piece of validity evidence was reported for all the questionnaires and most also had some reliability evidence, though there was no reported evidence of reliability for 4 of the questionnaires (Veronese and Pepe, 2013; Dokkedah et al., 2015; McDonald et al., 2019; Vinson and Chang, 2012). None of the questionnaires were evaluated for responsiveness.

**Table 3:** Quality appraisal results for the questionnaires included in the review.

	Reliability			Validity								Responsiveness
	Internal Consistency	Test-retest	Inter-rater	Content validity	Criterion-related validity		Construct validity					
					Concurrent validity	Predictive validity	Within-scale analyzes	Analyzes against external criteria				
								Convergent validity	Discriminant validity	Known group differences	Hypotheses testing	
AUDIT (Blair, 2017)	+++	••	••	••	••	••	++	••	••	++	••	••
CES-D (Getnet, 2019)	+++	••	••	+++	••	••	++	++	••	••	••	••
Community-based anger measure (Liddell, 2013)	••	••	••	••	+++	••	••	••	••	••	••	••
Culturally adapted checklist for complicated grief (later developed into the complicated bereavement module of the R-MHAP) (Tay, 2016)	+++	++	++	+++	••	••	+++	••	••	••	+	••
Complicated bereavement module of the R-MHAP (Tay, 2019)	+++	••	••	+++	••	••	+++	••	••	••	••	••
Culturally adapted checklist for PTSD and CPTSD (Tay, 2015)	++	+++	++	+++	••	••	++	++	••	••	+	••
CRIES-13 (Veronese, 2013)	+++	••	••	••	••	••	++	+	••	••	••	••
EPDS (Ing, 2017)	+	••	••	+	+++	••	+	••	••	••	••	••
GHQ-28 (Farhood, 2015)	+++	••	••	••	••	••	+	+++	••	••	••	••
HSCL-25 (Elsass, 2009)	+++	••	••	+	+	••	+	••	••	••	+	••
HSCL-25 – depression subscale (Bolton, 2001)	+++	+	••	••	++	••	+++	••	••	••	••	••
HSCL-25 (Trembley, 2009)	++	••	++	++	••	••	+	••	••	••	+	••
HSCL-25 (Ventevogel, 2007)		••	+++	+++	+	••	+	++	••	••	••	••
HTQ (DSM-4 version) (Michalopoulos, 2015)	+++	••	••	••	••	••	++	••	••	••	••	••
HTQ (DSM-5 version) (Michalopoulos, 2015)	+++	••	••	••	••	••	++	••	••	••	••	••
HTQ (Tay, Jayasuriya, et al., 2017)	••	+++	••	••	••	••	+++	+	••	••	+++	••
HTQ (Tay, Mohsin, et al., 2017)	+++	••	••	••	••	••	+++	••	••	++	+++	••
ICD-11 Trauma Questionnaire for CPTSD (Dokkedah, 2015)	••	••	••	••	••	••	••	++	••	+++	++	••
ICD-11 Truama Questionnaire for PTSD (Dokkedah, 2015)	••	••	••	••	0	••	••	++	••	+++	++	••
IES-R (Miller, 2009)	++	••	••	••	••	••	+	++	••	••	0	••
IES-R (Morina, 2010)	+++	••	••	••	••	••	++	••	••	••	••	••
IES-R (Morina, 2013)	+++	••	••	••	+++	••	+	••	••	••	••	••
International Trauma Questionnaires (Valliѐres, 2018)	+++	••	••	++	••	••	+	••	••	••	••	••
PCL-17-C (McDonald, 2019)	+++	••	••	••	••	••	+++	+++	••	••	+++	••
PCL-5 (Ibrahim, 2018)	+++	••	••	••	++	••	+	++	••	••	+	••
PRP-WPQ (Jayawickreme 2012)	+++	••	••	++	••	••	++	++	••	••	++	••
PTDS (Powell, 2005)	+++	••	••	••	••	••	+++	++	••	••	••	••
PTDS (Vinson, 2012)	••	••	••	••	••	••	+	••	••	••	••	••
PTSD and CPTSD R-MHAP modules (Silove, 2017)	••	••	••	••	••	••	++	+	••	••	••	••
PTSD and CPTSD R-MHAP modules (Tay, 2018)	+++	••	••	••	••	••	+	••	••	••	+++	••
RHS-15 (Fellmeth, 2018)	+	••	••	••	++	••	••	••	••	••	••	••
R-MHAP (Tay, 2015)	+++	••	••	+++	+++	••	••	••	••	••	••	••
Trauma Questionnaire (Trembley, 2009)	+++	••	++	++	••	••	+	••	••	••	+++	••

Grading system for acceptability: 0 = no evidence in favour, + = limited evidence in favour, ++ = moderate evidence in favour, +++ = strong evidence in favour, •• = no data available

AUDIT: Alcohol Use Disorders Identification Test, CES-D: Centre for Epidemiologic Studies Depression Scale; CPTSD: Complex posttraumatic stress disorder**;** CRIES-13: Children's Revised Impact of Events Scale-13; DSM: Diagnostic and Statistical Manual; EPDS: Edinburgh Postnatal Depression Scale**;** GHQ-28: General Health Questionnaire-28**;** HSCL-25: Hopkin's Symptom Checklist-25**;** HTQ: Harvard Trauma Questionnaire; ICD-11: International Classification of Disease-11; IES-R: Impact of Events Scale-Revised; PCL-17-C: Posttraumatic Stress Disorder Checklist – 17 – Civilian; PCL-5: Posttraumatic Stress Disorder Checklist for DSM-5; PRP-WPQ: The Penn/RESIST/Peradeniya War Problems Questionnaire; PTSD: Posttraumatic stress disorder; PTDS: Posttraumatic Stress Disorder Diagnostic Scale; R-MHAP: Refugee-Mental Health Assessment Package; RHS-15: Refugee Health Screener

Almost all questionnaires evaluated internal consistency and generally there was strong evidence for this. The other indicators of reliability were much less frequently evaluated with only 4 questionnaires reporting test-retest reliability and 5 for inter-rater reliability.

Content validity was relatively frequently assessed with moderate-strong evidence in favour overall. Overall, criterion-related validity was rarely assessed with moderate evidence in favour. Many study authors noted the difficulty of gathering data for a gold standard criterion for mental health constructs especially in conflict-affected low resource settings. Construct validity was mostly assessed using within-scale analyzes (although this produced variable quality of evidence), convergent validity or some other form of hypothesis testing. Notably responsiveness was not evaluated for any questionnaire.

For the 24 questionnaires that were adapted for use in new settings, the results of psychometric appraisal based on evidence from the original development papers (i.e. in the original setting) are presented in Table 4. Notably, a higher proportion asses test-retest reliability, some forms of construct validity and responsiveness. The quality of evidence reported in favour of these original development papers is also, on average, higher and more consistent in comparison to the results for the questionnaires adapted for use in conflict-affected settings.

**Table 4:** Quality appraisal results for the development papers for the adapted questionnaires included in the review (i.e. from the original setting*).

	Reliability			Validity								Responsiveness
	Internal Consistency	Test-retest	Inter-rater	Content validity	Criterion-related validity		Construct validity					
					Concurrent validity	Predictive validity	Within-scale analyzes	Analyzes against external criteria				
								Convergent validity	Discriminant validity	Known group differences	Hypotheses testing	
AUDIT	+++	+++	••	+++	+++	+++	••	+++	••	••	+	••
CES-D	+++	++	+	••	••	••	++	++	++	++	++	+++
CRIES-13	••	••	••	••	+++	••	++	++	••	••	••	••
EPDS	+++	+++	••	••	+++	••	••	••	••	••	••	+++
GHQ-28	••	••	••	••	+++	••	••	••	••	••	••	••
HSCL-25	••	+++	••	••	+++	••	••	+	••	••	••	••
HTQ	+++	+++	+++	+++	++	••	+++	••	++	••	++	••
ICD-11 Trauma Questionnaire for CPTS	••	••	••	••	••	••	++	++	++	••	••	••
ICD-11 Trauma Questionnaire for PTSD	••	••	••	••	••	••	++	++	++	••	••	••
IES-R	+++	+++	••	••	••	••	+++	+++	••	••	••	••
International Trauma Questionnaires	••	••	••	••	••	••	+++	••	••	••	••	••
PCL-17- C	+++	+++	••	••	••	••	+++	+++	+++	••	++	••
PCL-5	+++	+++	••	••	••	••	++	+++	+++	+	+++	••
PTDS	+++	+++	••	++	+++	••	++	+++	••	••	••	••
RHS-15	+++	••	••	+++	+++	••	••	+++	••	••	••	••

Grading system for acceptability: 0 = no evidence in favour, + = limited evidence in favour, ++ = moderate evidence in favour, +++ = strong evidence in favour, •• = no data available

AUDIT: Alcohol Use Disorders Identification Test, CES-D: Centre for Epidemiologic Studies Depression Scale; CPTSD: Complex posttraumatic stress disorder**;** CRIES-13: Children's Revised Impact of Events Scale-13; DSM: Diagnostic and Statistical Manual; EPDS: Edinburgh Postnatal Depression Scale**;** GHQ-28: General Health Questionnaire-28**;** HSCL-25: Hopkin's Symptom Checklist-25**;** HTQ: Harvard Trauma Questionnaire; ICD-11: International Classification of Disease-11; IES-R: Impact of Events Scale-Revised; PCL-17-C: Posttraumatic Stress Disorder Checklist – 17 – Civilian; PCL-5: Posttraumatic Stress Disorder Checklist for DSM-5; PRP-WPQ: The Penn/RESIST/Peradeniya War Problems Questionnaire; PTSD: Posttraumatic stress disorder; PTDS: Posttraumatic Stress Disorder Diagnostic Scale; R-MHAP: Refugee-Mental Health Assessment Package; RHS-15: Refugee Health Screener

⁎ These quality appraisal results are solely based on the evidence presented in the development papers for the adapted questionnaires included in the review to allow for comparison between the evidence reported in the original settings (often non-conflict-affected) and the evidence for the questionnaires adapted for use in conflict-affected settings (as presented in Table 3)

This review included 30 studies which reported measurement properties from 33 unique questionnaires. There was high variability in the range of measurement properties reported and the quality of questionnaires. Overall, for the measurement properties reported, there was moderate evidence for reliability and validity, although there were many gaps in the availability of data.

## 4 Discussion

Our findings show the growth of publications in this area over the past two decades, reflecting those of other systematic reviews on mental health among conflict-affected populations in LAMICS. (Charlson et al., 2019) There has also been increasing recognition of the particular importance of psychometrics in this field to facilitate the development of good quality questionnaires that can be administered by non-specialists in LAMICs. (Rasmussen and Jayawickreme, 2020)

However, gaps remain. There were few studies involving IDPs despite there being almost twice as many IDPs as refugees globally. In terms of outcomes, the eligible studies mostly focus on PTSD, depression or anxiety and neglect other serious mental illnesses such as psychotic disorders, alcohol disorder and other substance misuse disorders. In addition, the vast majority of the study authors were from HICs adding weight to concerns expressed elsewhere about the inequitable authorship in research with conflict-affected populations in LAMICs. (Sibai et al., 2019; Siriwardhana et al., 2011).

There was variation in the evidence presented for different measurement properties. Internal consistency was frequently reported with strong evidence but this does not necessarily constitute sufficient evidence of reliability. (U. S. Food and Drug Administration Center for Biologics Evaluation and Research, 2006) The majority of studies did not assess content validity and, of those studies that tested for content validity, most studies did not present a conceptual framework reflecting findings elsewhere in refugee research that there is a lack of theoretical bases to questionnaires. (Hollifield et al., 2002) This is an important finding as lack of clarity about the construct that is being measured will reduce the extent to which other psychometric properties can be demonstrated. An instrument without a clear conceptual underpinning is therefore less likely to be robust.

No studies reported on responsiveness or predictive validity. Given that that the purpose for most of these questionnaires included is discriminative (i.e. to detect mental health disorders as part of a prevalence survey) rather than evaluative or predictive, these measurement properties are perhaps less relevant depending on the intended use of the questionnaire. However, if a questionnaire is intended to detect clinically meaningful change (i.e. for evaluation of an intervention) then responsiveness needs to be established to ensure that the questionnaire is fit for purpose.

We did not find a clear distinction in quality between newly developed questionnaires and the questionnaires adapted for use in new settings. For the questionnaires adapted in multiple different settings (e.g. the HSCL-25) there was not strong consistency in the measurement properties recorded across different settings. For the adapted questionnaires, the quality appraisal results were slightly weaker in comparison to the results from the quality appraisal results for the original development papers, providing weak evidence that the quality of questionnaires in conflict-affected settings is lower than in non-conflict-affected settings.

The availability of data makes it difficult to truly understand the differences in quality between newly developed and adapted questionnaires or the different properties for the same questionnaire adapted in multiple different settings. Appraising the quality of the psychometric data was also made difficult by variations in psychometric nomenclature and reporting standards as has been found by psychometric reviewers in other fields. (Mokkink et al., 2010) Included studies also frequently referenced data for measurement properties from questionnaires validated in different settings, which made it difficult to apply strict psychometric criteria.

There are clearly many logistical, methodological and ethical constraints in conducting research on mental health in conflict-affected settings. Designing and conducting a high-quality validation study is a lengthy process that requires highly skilled personnel and adequate long-term funding. These are not requirements that necessarily fit well with the resources available in conflict-affected settings. (Blanchet et al., 2017) The challenge lies in finding the balance between generating adequate quality and utility of evidence for questionnaire-based studies on mental disorders whilst working within resource constraints.

### 4.1 Recommendations

The results from this review suggest that the most pressing priorities are to: (i) conduct research equitably with more involvement of researchers from LAMICs and involving a broader range of affected populations (particularly IDPs); (ii) emphasise the need to develop a conceptual framework and fully test content validity as part of the process of developing a new questionnaire; (iii) improve reporting standards, including clearly stating the intended purpose for questionnaires and reporting measurement properties accordingly; (iv) encourage more thorough testing of reliability instead of relying solely on internal consistency; (v) establish appropriate methods for criterion-related validity when there are inadequate resources for establishing the diagnosis through clinical interview and; (vi) strengthen capacity in LAMICs for the use of such methods*.*

Mental health services for conflict-affected populations in LAMICs are often co-ordinated by humanitarian agencies who need adequate mental health data to guide service provision. The key policy implications from the results of this review for such humanitarian agencies and other services providers are to: (i) scrutinise the quality of the mental health questionnaires used to inform decision-making processes (ii) acknowledge the limitations of the data gathered by such measures (iii) define the acceptable limits for the quality of mental health measures according to the nature of the decision(s) to be made based on the data gathered and; (iv) invest adequate resources into development work for mental health measures to allow for the collection of adequate data.

### 4.2 Limitations

Limitations for this review include that only English and French papers were included which is likely to have missed relevant data from other languages. The identification of 5 extra articles for inclusion by manual searching indicates that, despite the broad scope of the search terms, further studies may also have been missed. Questionnaires for general psychological health and mental distress, including locally derived outcomes, were excluded as the focus of this review was on diagnostic instruments to allow for comparisons to be made across settings although we acknowledge that this limits the scope of this review.

## 5 Conclusion

This systematic review assessed the quality of mental health questionnaires that have either been developed or validated in conflict-affected settings in LAMICS. It highlighted the limited quantity and quality of questionnaires. Key priorities are to: improve equity in authorship and populations covered; strengthen research capacity on this topic; and stronger use of conceptual frameworks and reporting standards to allow future users of the questionnaires to more easily discern whether the questionnaires are appropriate for use with other conflict-affected populations.

## CRediT authorship contribution statement

**Sharon Christy:** Conceptualization, Visualization, Data curation, Investigation, Writing – original draft. **Chesmal Siriwardhana:** Conceptualization, Visualization. **Julia Lohmann:** Investigation, Writing – review & editing. **Bayard Roberts:** Conceptualization, Visualization, Writing – review & editing. **Sarah Smith:** Conceptualization, Visualization, Supervision, Writing – review & editing.

## Declarations of Competing Interest

None.

## Appendix A Search terms

CINAHL Plus with Full Text

1 (MH "Research Measurement") OR (MH "Outcome Assessment") OR (MH "Outcomes Research")
2 (MH "Mental Disorders+") OR "mental disorders"
3 (MH "Research, Mental Health") OR (MH "Mental Health Screening (Saba CCC)")
4 (mental OR psychiatr*) AND (health OR illness OR disorder)) OR dementia OR alzheimers OR "alcohol disorder" or "substance disorder" or "drug disorder" OR psycho* OR schizo* OR delusion* OR "mood disorder" OR "affective disorder" OR depressi* OR mania OR bipolar OR anxiety OR PTSD OR "post-traumatic stress disorder"
5 2 OR 3 OR 4
6 (MH "War+") OR "war"
7 (MH "Refugees") OR "refugee"
8 war OR "conflict zone" OR "war-zone" OR "conflicted-affected" OR "war-affected" OR refugee* OR "asylum seeker" OR "internally displaced people" OR "externally displaced people"
9 6 OR 7 OR 8
10 1 AND 5 AND 9

Embase

1 exp questionnaire/
2 exp psychometry/
3 exp outcome assessment/
4 exp psychological rating scale/ or exp psychologic assessment/
5 exp reliability/ or exp validity/
6 1 or 2 or 3 or 4 or 5
7 exp mental disease/
8 ((mental or psychiatric) adj3 (health or illness$ or disorder$)).mp.
9 (dementia or alzheimer$).mp
10 ((substance or alcohol or drug) adj3 (abuse or disorder$ or addiction or dependence or misuse)).mp.
11 (psychosis or psychotic or schizo$ or delusion$).mp
12 (((mood or affective) adj3 disorder) or depression or depressive or manic depression or mania or bipolar).mp.
13 ((((anxiety or PTSD or post-traumatic stress disorder or panic) adj3 (disorder$ or attack$)) or phobia or stress) adj3 (reaction or disorder)).mp.
14 7 or 8 or 9 or 10 or 11 or 12 or 13
15 exp war/
16 exp ethnic conflict/
17 exp refugee/
18 exp asylum seeker/
19 ((conflict-affected or warzone or war-zone or (war or conflict)) adj3 (affected or induced or zone)).mp
20 ((displaced adj3 (internally or people or persons)) or IDP$ or refugee$ or asylum seeker).mp
21 15 or 16 or 17 or 18 or 19 or 20
22 6 and 14 and 21
23 limit 22 to (english or french)
24 limit 23 to human

Global Health

1 diagnosis.sh.
2 exp questionnaires/
3 screening.sh.
4 exp validity/
5 exp reliability/
6 exp factor analysis/
7 (valid or reliab*).mp.
8 1 or 2 or 3 or 4 or 5 or 6 or 7
9 exp mental disorders/
10 (depression or mental health or anxiety or schizophrenia or psychoses).sh.
11 ((mental or psychiatric) adj3 (health or illness$ or disorder$)).mp. [mp=abstract, title, original title, broad terms, heading words, identifiers, cabicodes]
12 (dementia or alzheimer$).mp.
13 ((substance or alcohol or drug) adj3 (abuse or disorder$ or addiction or dependence or misuse)).mp.
14 (psychosis or psychotic or schizo$ or delusion$).mp
15 (((mood or affective) adj3 disorder) or depression or depressive or manic depression or mania or bipolar).mp.
16 (((anxiety or PTSD or post-traumatic stress disorder or panic) adj3 (disorder$ or attack$)) or phobia or (stress adj3 (reaction or disorder))).mp
17 9 or 10 or 11 or 12 or 13 or 14 or 15 or 16
18 exp conflict/
19 exp war/
20 exp refugees/
21 ((conflict-affected or warzone or war-zone or (war or conflict)) adj3 (affected or induced or zone)).mp.
22 ((displaced adj3 (internally or people or persons)) or IDP$ or refugee$ or asylum seeker).mp.
23 18 or 19 or 20 or 21 or 22
24 8 and 17 and 23

Ovid MEDLINE(R)

1 (instrumentation or methods).sh.
2 ("validation studies" or "comparative study").pt.
3 exp Psychometrics/
4 (psychometr* or clinimetr* or clinometr*).tw.
5 exp "Outcome Assessment (Health Care)"/
6 ("outcome assessment" or "outcome measure" or "observer variation").tw.
7 exp Observer Variation/
8 exp Health Status Indicators/
9 exp "Reproducibility of Results"/
10 reproducib*.tw.
11 exp Discriminant Analysis/
12 (reliab* or unreliab* or valid* or "coefficient of variation" or coefficient or homogeneity or homogeneous or "internal consistency").tw.
13 (cronbach* and (alpha or alphas)).tw.
14 (item and (correlation* or selection* or reduction*)).tw.
15 (agreement or precision or imprecision or "precise values" or test-retest).tw.
16 (test and retest).tw.
17 (reliab* and (test or retest)).tw.
18 (stability or interrater or inter-rater or intrarater or intra-rater or intertester or inter-tester or intratester or intra-tester or interobserver or inter-observer or intraobserver or intra-observer or intertechnician or inter-technician or intratechnician or intra-technician or interexaminer or inter-examiner or intraexaminer or intra-examiner or interindividual or inter-individual or intraindividual or intra-individual or interparticipant or inter-participant or intraparticipant or intra-participant).tw.
19 (kappa or kappa's or kappas or repeatab*).tw.
20 ((replicab* or repeated) and (measure or measures or findings or result or results or test or tests)).tw.
21 (generali#za* or concordance).tw.
22 (intraclass and correlation).tw.
23 (discriminative or "known group" or "factor analysis" or "factor structure" or "factor structures" or dimension or subscale*).tw.
24 (multitrait and scaling and analys#s).tw.
25 ("item discriminant" or "interscale correlation" or error or errors or "individual variability" or "interval variability" or "rate variability").tw.
26 (variability and (analysis or values)).tw.
27 (uncertainty and (measurement or measuring)).tw.
28 ("standard error of measurement" or sensitivity or responsive*).tw.
29 (limit and detection).tw.
30 ("minimal detectable concentration" or interpretab*).tw.
31 ((minimal or minimally or clinical or clinically) and (important or significant or detectable) and (change or difference)).tw.
32 (small and (real or detectable) and (change or difference)).tw.
33 ("meaningful change" or "ceiling effect" or "floor effect" or "item response model" or irt or rasch or "differential item functioning" or dif or "computer adaptive testing" or "item bank" or "cross-cultural equivalence").tw.
34 1 or 2 or 3 or 4 or 5 or 6 or 7 or 8 or 9 or 10 or 11 or 12 or 13 or 14 or 15 or 16 or 17 or 18 or 19 or 20 or 21 or 22 or 23 or 24 or 25 or 26 or 27 or 28 or 29 or 30 or 31 or 32 or 33
35 (adresses or biography or "case reports" or comment or directory or editorial or festschrift or interview or lectures or "legal cases" or legislation or letter or news or "newspaper article" or "patient education handout" or "popular works" or congresses or "consensus development conference" or "consensus development conference, nih" or "practice guideline").pt.
36 Animals/
37 35 or 36
38 34 not 37
39 exp Mental Disorders/
40 ((mental or psychiatric) adj3 (health or illness* or disorder*)).tw.
41 (dementia or alzheimer*).tw.
42 ((substance or alcohol or drug) adj3 (abuse or disorder* or addiction or dependence or misuse)).tw.
43 (psychosis or psychotic or schizo* or delusion*).tw.
44 (((mood or affective) adj3 disorder) or depression or depressive or manic depression or mania or bipolar).tw.
45 ((((anxiety or PTSD or post-traumatic stress disorder or panic) adj3 (disorder* or attack*)) or phobia or acute stress) adj3 (reaction or disorder)).tw.
46 39 or 40 or 41 or 42 or 43 or 44 or 45
47 exp Refugees/
48 exp Warfare/
49 ((conflict-affected or warzone or war-zone or (war or conflict)) adj3 (affected or induced or zone)).mp.
50 (post-conflict or after-conflict or ((post or after) adj3 (war or conflict))).mp.
51 ((displaced adj3 (internally or people or persons)) or IDP* or refugee* or "asylum seeker*").mp.
52 47 or 48 or 49 or 50 or 51
53 38 and 46 and 52
54 limit 53 to (english or french)
55 limit 54 to humans

PsycINFO

1 exp Inventories/ or exp Test Construction/ or exp Questionnaires/ or exp Rating Scales/ or exp Test Reliability/ or exp Measurement/ or exp Psychometrics/ or exp Test Validity/ or exp Criterion Referenced Tests/ or exp Foreign Language Translation/
2 (valid* or reliab*).mp.
3 1 or 2
4 exp Mental Disorders/
5 exp PSYCHOSIS/
6 exp Affective Disorders/
7 exp ANXIETY DISORDERS/
8 ((mental or psychiatric) adj3 (health or illness$ or disorder$)).mp
9 (dementia or alzheimer$).mp.
10 ((substance or alcohol or drug) adj3 (abuse or disorder$ or addiction or dependence or misuse)).mp.
11 (psychosis or psychotic or schizo$ or delusion$).mp.
12 (((mood or affective) adj3 disorder) or depression or depressive or manic depression or mania or bipolar).mp
13 (((anxiety or PTSD or post-traumatic stress disorder or panic) adj3 (disorder$ or attack$)) or phobia or (stress adj3 (reaction or disorder))).mp.
14 4 or 5 or 6 or 7 or 8 or 9 or 10 or 11 or 12 or 13
15 exp WAR/
16 exp REFUGEES/
17 ((conflict-affected or warzone or war-zone or (war or conflict)) adj3 (affected or induced or zone)).mp.
18 ((displaced adj3 (internally or people or persons)) or IDP$ or refugee$ or asylum seeker).mp
19 (post-conflict or after-conflict or ((post or after) adj3 (war or conflict))).mp.
20 15 or 16 or 17 or 18 or 19
21 3 and 14 and 20
22 limit 21 to (english or french)
23 limit 22 to human
